# Derailing individualized ovarian stimulation

**DOI:** 10.1093/humrep/dey060

**Published:** 2018-02-08

**Authors:** Scott M Nelson, Richard A Anderson

**Affiliations:** 1 School of Medicine, University of Glasgow, Glasgow G31 2ER, UK; 2 MRC Centre for Reproductive Health, Queens Medical Research Institute, University of Edinburgh, Edinburgh EH16 4TJ, UK

Sir,

We read with interest the paper by Tilborg that incorporated a *post hoc* analysis of the utility of anti-müllerian hormone (AMH) for a cost-effectiveness analysis (van Tilborg *et al.*, 2017). The authors predicated the randomized control trial (RCT) and individualization of dose solely on antral follicle count, but there are a number of substantial issues that question the ability to retrospectively draw conclusions on the utility of AMH to individualize treatment. Specifically, longitudinal measurement of serum AMH shows during GnRH-agonist downregulation shows marked and clinically relevant changes, an effect dependent on the duration of GnRH-agonist treatment (Su *et al.*, 2013, Drakopoulos *et al.*, 2017). Therefore, serum AMH level should be measured before start of GnRH-agonist downregulation and not once started, as performed by Tilborg and colleagues. Secondly, four AMH categories have been developed, but is unclear what modelling underlies these thresholds. Particularly AMH of 1.33 to <2.25ng/ml (9.5–18.0 pmol/l) was used to classify normal responders, but in recent phase II (Arce *et al.*, 2014) and phase III RCTs (Nyboe Andersen *et al.*, 2017), a poor response to ovarian stimulation was anticipated with AMH values <15.0 pmol/l (<2.1 ng/ml) (Arce *et al.*, 2014). Thirdly, retrospectively predicting the outcome of individualization of treatment based on AMH assumes equivalence with AFC in their association with oocyte yield. In contrast to older meta-analyses, several recent multicentre studies demonstrate that AMH exhibits an almost two-fold higher correlation coefficient with oocyte yield than that observed for AFC determined across multiple sites (Nelson *et al.* 2015) and substantially lower variability (Anderson *et al.*, 2015). The variability between trial sites in AFC (or indeed in AMH) for the current study is not presented. Lastly, the authors use statistical methodology to predict the outcome for the 24% of women where AMH and AFC were discordant. However, this model incorporates the applied FSH dose which was based on the AFC but not on AMH, and the assumption that *post hoc* modelled predictions can replace real clinical outcomes is questionable. Collectively, these concerns would suggest that, rather than retrospectively infer conclusions about the utility of AMH for individualizing treatment from incorrectly timed samples, evidence from a large scale prospective international multicentre RCT should be used (Nyboe Andersen *et al.*, 2017).
